# Interpretation of Mendelian randomization using a single measure of an exposure that varies over time

**DOI:** 10.1093/ije/dyac136

**Published:** 2022-07-15

**Authors:** Tim T Morris, Jon Heron, Eleanor C M Sanderson, George Davey Smith, Vanessa Didelez, Kate Tilling

**Affiliations:** MRC Integrative Epidemiology Unit at the University of Bristol, Bristol, UK; Population Health Sciences, Bristol Medical School, University of Bristol, Bristol, UK; MRC Integrative Epidemiology Unit at the University of Bristol, Bristol, UK; Population Health Sciences, Bristol Medical School, University of Bristol, Bristol, UK; MRC Integrative Epidemiology Unit at the University of Bristol, Bristol, UK; Population Health Sciences, Bristol Medical School, University of Bristol, Bristol, UK; MRC Integrative Epidemiology Unit at the University of Bristol, Bristol, UK; Population Health Sciences, Bristol Medical School, University of Bristol, Bristol, UK; Leibniz Institute for Prevention Research and Epidemiology—BIPS, Bremen, Germany; Department of Mathematics and Computer Science, University of Bremen, Bremen, Germany; MRC Integrative Epidemiology Unit at the University of Bristol, Bristol, UK; Population Health Sciences, Bristol Medical School, University of Bristol, Bristol, UK

**Keywords:** Mendelian randomization, causal inference, longitudinal, simulation

## Abstract

**Background:**

Mendelian randomization (MR) is a powerful tool through which the causal effects of modifiable exposures on outcomes can be estimated from observational data. Most exposures vary throughout the life course, but MR is commonly applied to one measurement of an exposure (e.g. weight measured once between ages 40 and 60 years). It has been argued that MR provides biased causal effect estimates when applied to one measure of an exposure that varies over time.

**Methods:**

We propose an approach that emphasizes the liability that causes the entire exposure trajectory. We demonstrate this approach using simulations and an applied example.

**Results:**

We show that rather than estimating the direct or total causal effect of changing the exposure value at a given time, MR estimates the causal effect of changing the underlying liability for the exposure, scaled to the effect of the liability on the exposure at that time. As such, results from MR conducted at different time points are expected to differ (unless the effect of the liability on exposure is constant over time), as we illustrate by estimating the effect of body mass index measured at different ages on systolic blood pressure.

**Conclusion:**

Univariable MR results should not be interpreted as time-point-specific direct or total causal effects, but as the effect of changing the liability for the exposure. Estimates of how the effects of a genetic variant on an exposure vary over time, together with biological knowledge that provides evidence regarding likely effective exposure periods, are required to interpret time-point-specific causal effects.

Key Messages

- Many exposures of interest vary over time, yet Mendelian randomization (MR) is commonly applied to one measurement of an exposure.
- MR does not estimate direct or total causal effect of changing an exposure value at a given time.
- MR estimates the causal effect of changing the exposure liability that gives rise to an exposure value at a given time.
- MR results from time-varying exposures should be interpreted with respect to the underlying liability for an exposure.

## Introduction

### Mendelian randomization

Mendelian randomization (MR) is a powerful tool through which the causal effects of modifiable exposures (risk factors) can be estimated from observational data under assumptions that in some circumstances may be more plausible than the unmeasured confounding and no measurement error assumptions required by conventional methods.^1^ MR is generally implemented within an instrumental variables (IV) framework that exploits the randomization inherent in the allocation of genotypes at conception and gamete cell formation, using this random variation in alleles to instrument differences in observed exposures between individuals.^2^ Reverse and residual confounding is reduced because the formation of the genotype occurs prior to the phenotypic development and is generally not related to environmental factors.^3^^,^^4^

Three assumptions are required for MR analyses to test the null hypothesis that an exposure X does not cause an outcome Y for any individuals. These are (i) relevance: that genotype is associated with the exposure of interest; (ii) independence: that there is no common cause of genotype and outcome; (iii) exclusion: that genotype does not affect the outcome through any path other than the exposure.^5^^,^^6^ In order to estimate an average treatment effect, extra assumptions are needed: first, that a difference in average exposure between populations with and without a risk allele would result in the same difference in outcome as if an environmental factor increased average exposure in a population by the same amount (gene–environment equivalence) and, second, that the structural model relating the exposure and outcome is linear and additive with a homogeneous effect of the exposure on the outcome.^7–9^

MR studies have largely leveraged information from a single measurement of the exposure and outcome, often due to limited availability of repeatedly measured data. Many exposures of interest vary over time,^10^^,^^11^ being subject to both between-individual and within-individual variation. Within-individual variation may be largely a function of measurement error (e.g. height in adulthood^12^), longitudinal within-individual phenotypic variability (BMI^13^), monotonic change (myopia^14^) or, likely, a mixture of these. Time-varying genetic associations have been reported for a range of phenotypes,^15–21^ thus consistent effect sizes may not be estimated when applying MR to exposures measured at different time points across the lifecourse, regardless of sampling variation and measurement error.^18^^,^^22^

### MR applied to one measure of an exposure that varies over time

It has long been recognized that MR estimates relate to exposures that generally act over a considerable period of time, often since birth.^1^^,^^23^ Many single-nucleotide polymorphism (SNP)-phenotype associations have a consistent direction of effect and similar effect sizes throughout the lifecourse, though this pattern is not uniform.^24^ More recently, it has been questioned how appropriate MR is when applied to exposures that vary over time.^25–27^ Labrecque and Swanson propose one possible definition of a lifetime effect that might be of interest: the effect of increasing the exposure by one unit at each time point throughout the lifecourse.^25^ In order to estimate this effect using MR, the association between genotype and exposure needs to be constant over time, giving rise to parallel exposure trajectories (a one-unit change in the genotype will give rise to the same difference in exposure at time t as at time t + 1). They demonstrated that estimates of this causal effect from MR differ over time in the presence of time-varying genotype–exposure associations, concluding that MR provides a biased estimate of the causal effect of increasing the exposure by one unit at each time point. Concerns have also been raised that MR with time-varying exposures may be biased if a feedback mechanism exists in which genetic factors influence predisposition to an outcome, which in turn influences the exposure at a subsequent time point.^26^ For example, where instruments for coronary heart disease (CHD) relate to C-reactive protein (CRP) because the instruments for CHD relate to developing atheroma, which in turn increases CRP.

**Figure 1:**
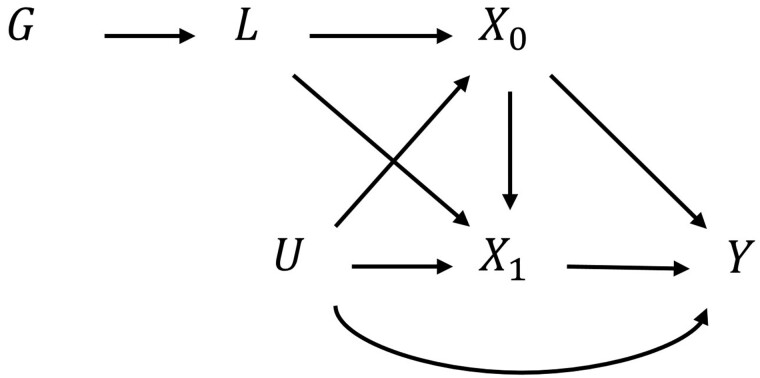
Causal diagram showing two exposures and one outcome G , genetic instrument; L, liability; $X_{0}$, exposure measured at time 0; $X_{1}$, exposure measured at time 1; Y, outcome; U, confounder. Other sources of variability in the liability, exposures and outcome are not shown in this diagram. There is a problem of under-identification here in that the direct effects of $X_{0}$ or $X_{1}$ on Y cannot be estimated with a single liability (L).

We propose an approach that uses MR to assess the effect on the outcome of the change in the entire exposure history that would be induced by a change in genotype. That is, we are not estimating the causal effect of an exposure as it manifests at a given time point, but the effect of the underlying exposure liability. That is, we assume that there is some unobserved (latent) variable L, which is caused by the genotype G, and in turn causes the exposure at every instance across the lifecourse. Although the effect of liability on outcome is the estimand of interest, the liability is unobserved, so we must estimate its effect via the measured exposure(s). Here, we consider the simplified case with one genetic instrument (G), a time-varying continuous exposure that only occurs on two occasions ($X_{0}$ and $X_{1}$), one of which is measured, an outcome that occurs at one time point (Y) and an unmeasured confounder U (Figure 1). Thus, a change in genotype changes L, which changes both $X_{0}$ and $X_{1}$. The case in which X occurs in continuous time is described in the Supplementary text (available as Supplementary data at *IJE* online).

Our approach overcomes two problems with the interpretation of MR with time-varying exposures. First, if G changes, both $X_{0}$ and $X_{1}$ must be changed *together*; a one-unit change in G (e.g. an increase of one risk allele) cannot change one of the exposures in isolation. Where time-varying genetic effects exist, the change in genotype G required to raise a given exposure trajectory by one unit at time t (e.g. raising the weight by 1 kg at birth) may be quite different to the change in genotype required to change the exposure by one unit at time t + k (e.g. raising the weight by 1 kg at age 50 years). Second, a one-unit change in G cannot have an arbitrary effect on the exposure trajectory (e.g. increasing exposure by exactly one unit at all times). Thus, univariable MR with one genetic instrument that acts on exposure over a period of time cannot be used to recover the effect of a change in exposure at a single time, nor of *any arbitrary* change to the trajectory of exposure. Instead, we argue that MR with a time-varying continuous exposure can be used to examine the effect of a *specific* change in the trajectory of that exposure, depending on how the genotype impacts the trajectory. Here, the effect refers to the liability L, i.e. we are estimating the effect on the outcome of changing L. It should be noted that there will be no information about the shape of the typical trajectory from a study with only one measurement of a time-varying exposure taken at the same time point for all participants. In this case external information would be needed (e.g. from a separate longitudinal study).

This interpretation of the MR estimate can be clearly seen from the terms in the Wald estimator. Here, the numerator is the effect of a change in SNP on the outcome. The denominator is the effect of a change in SNP on the exposure at a given time point. This denominator is equal to the effect of a one-unit change in SNP on the liability, multiplied by the total effect of the liability on the exposure at the given time point. Thus, the Wald estimate is the effect of a change in the liability on the outcome (effect of SNP on outcome divided by effect of SNP on exposure liability), scaled such that the liability causes a one-unit change in exposure at that time point.

For simplicity, our example has just one SNP causing L. However, L may be instrumented by multiple SNPs. The emphasis here is that the effects of $X_{0}$ and $X_{1}$ cannot be separated in the case where our instrument(s) act through a 1D liability (L). The effects of $X_{0}$ and $X_{1}$ could potentially be separately estimated within a multivariable MR framework if two or more different liabilities have been identified that have different effects on $X_{0}$ and $X_{1}$, and if there were really only two exposures.^28^ In more realistic scenarios, however, it is unlikely that the exposure will only occur at a small number of time points—it is likely to occur over continuous time (see Supplementary material, available as Supplementary data at *IJE* online).

In this paper, we clarify the causal quantities that are estimated by MR when applied to time-varying exposures with time-varying genetic effects assuming a liability structure and how they should be interpreted.

## Methods

### Effects of interest

We define two estimands of interest: the total effect of a one-unit change in an exposure $X_{k}$ (i.e. exposure X measured at a specific time point $t_{k}$) on an outcome Y ($\beta_{Tk}$); and a ‘liability effect’—the causal effect on Y of a change in the liability L, such that $X_{k}$ increases in expectation by one unit ($\beta_{L_{k}}$). We derive algebraic expressions for these estimands under a linear model in the case of a time-varying exposure that occurs at two time points and one outcome, with more general derivations given in the Supplementary material (available as Supplementary data at *IJE* online).

#### Total effect

We define $\beta_{Tk}$ to be the total effect of $X_{k}$ on the outcome Y, i.e. the change in Y from increasing $X_{k}$ by one unit. This includes the direct effect of $X_{k}$ on Y, and the indirect effect via the effect of $X_{k}$ on subsequent occurrences of the exposure $X_{m}$ where m > k.

#### Lifetime liability effect for a specific liability

We define the liability effect ($\beta_{L})\;$as the causal effect on Y of increasing the liability L by one unit. We define the liability effect at time *k* ($\beta_{L_{k}})\;$as the causal effect of changing the liability L such that the expected value of the exposure measured at time k is increased by one unit. This can be thought of as the effect of moving all individuals from the liability L giving rise to E(X) = x at time k, to a liability $L_{1}$ that would give rise to E(X) = x + 1 at time k.

**Figure 2:**
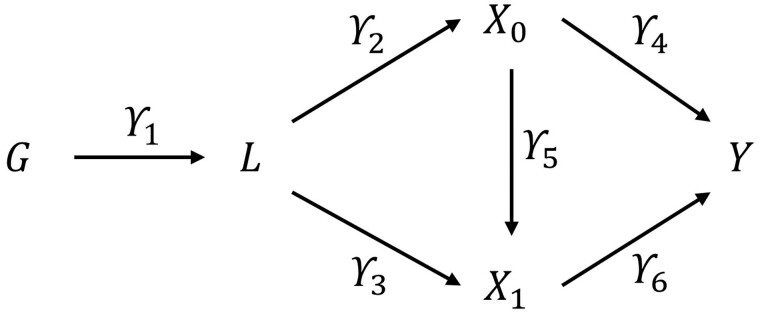
Directed acyclic graph showing the exposure liability in the context of two exposures and one outcome G , genetic instrument; L, liability; $X_{0}$, exposure measured at time 0; $X_{1}$, exposure measured at time 1; Y, outcome. Note that confounders or other causes of variability in liability, exposures or outcome are not shown.

We now derive expressions for the total and liability causal effect in the situation with an outcome Y that is caused by a genetically influenced liability *L* for an exposure X occurring at two time points ($X_{0}$ and $X_{1}$) (Figure 2). The genetic instrument G can have a non-linear relationship with the underlying liability L, but for simplicity we assume here that L is a linear function of G and U. We assume linearity and additivity from L to the exposure measurements $X_{k}$ and from exposures to outcome Y. The effect of L on exposure measures is allowed to change with time/age, thus the shape of the trajectory of X with age can be non-linear.

We assume (Figure 2) that:

$$L=\gamma_{1}G+\alpha_{L}U+\varepsilon_{L}$$

$$X_{0}=\gamma_{2}L+\alpha_{0}U+\varepsilon_{0}$$

$$X_{1}=\gamma_{3}L+\gamma_{5}X_{0}+\alpha_{1}U+\varepsilon_{1}$$

$$Y=\gamma_{6}X_{1}+\gamma_{4}X_{0}+\alpha_{Y}U+\varepsilon_{Y}$$

where all errors ($\varepsilon_{L},\varepsilon_{0},\;\varepsilon_{1}\;\mathrm{and}\;\varepsilon_{Y})$ are distributed with mean zero and are mutually independent.

The *total effect* of a one-unit change in $X_{0}$ on Y ($\beta_{T_{0}}$) is given by:

$$\beta_{T_{0}}=\gamma_{4}+\gamma_{5}\gamma_{6}$$

The *total effect* of a one-unit change in $X_{1}$ on Y is given by:

$$\beta_{T_{1}}=\gamma_{6}$$

The liability effect is the causal effect of a one-unit increase in liability, which is given by:

$${\beta_{L}=\gamma }_{2}\gamma_{4}+\gamma_{2}\gamma_{5}\gamma_{6}+\gamma_{3}\gamma_{6}$$

Turning to the liability effect at time 0, $\beta_{L_{0}}$ (the effect of increasing the liability such that $X_{0}$ increases in expectation by one unit), a one-unit increase in $E(X_{0})$ occurs because there is an increase in L from $l_{10}$ to L  $=\;l_{10}+\frac{1}{\left(\gamma_{2}\right)}$.

If ${L\;=\;l}_{10}$, then:

$$E\left(Y|do\left(l_{10}\right)\right)=y_{00}=l_{10}\left(\gamma_{2}\gamma_{4}+\gamma_{2}\gamma_{5}\gamma_{6}+\gamma_{3}\gamma_{6}\right)$$

If ${L=l}_{10}+\frac{1}{\left(\gamma_{2}\right)},\;$then:

$$E({\left(Y|do\left(l_{10}+\frac{1}{\left(\gamma_{2}\right)}\right)\right))=y}_{10}=\left(l_{10}+\frac{1}{\gamma_{2}}\right)\left(\gamma_{2}\gamma_{4}+\gamma_{2}\gamma_{5}\gamma_{6}+\gamma_{3}\gamma_{6}\right)$$

The effect on Y of changing the liability L such that it raises $X_{0}$ by one unit is therefore given by:

(1) $$\beta_{L_{0}}=y_{10}-{y_{00}}_{}=\frac{\left(\gamma_{2}\gamma_{4}+\gamma_{2}\gamma_{5}\gamma_{6}+\gamma_{3}\gamma_{6}\right)}{\gamma_{2}}$$

A one-unit increase in expectation in $X_{1}$ would occur because there is an increase in $L_{}$ from $l_{11}$ to $l_{11}+\frac{1}{\left(\gamma_{2}\gamma_{5}+\gamma_{3}\right)}$

If ${L_{}=l}_{11}$ then

$$E\left(Y|do\left(l_{11}\right)\right)=y_{01}=l_{11}\left(\gamma_{2}\gamma_{4}+\gamma_{2}\gamma_{5}\gamma_{6}+\gamma_{3}\gamma_{6}\right)$$

If ${L_{}=l}_{11}+\frac{1}{\left(\gamma_{2}\gamma_{5}+\gamma_{3}\right)}\;$then

$$E({\left(Y|do\left(l_{11}+\frac{1}{\left(\gamma_{2}\gamma_{5}+\gamma_{3}\right)}\right)\right))}_{}{=y}_{11}=\left(l_{11}+\frac{1}{\left(\gamma_{2}\gamma_{5}+\gamma_{3}\right)}\right)\left(\gamma_{2}\gamma_{4}+\gamma_{2}\gamma_{5}\gamma_{6}+\gamma_{3}\gamma_{6}\right)$$

The effect on Y of changing L such that $X_{1}$ is increased by one unit in expectation is given by:

(2) $$\beta_{L_{1}}=y_{11}-{y_{01}}_{}=\frac{\left(\gamma_{2}\gamma_{4}+\gamma_{2}\gamma_{5}\gamma_{6}+\gamma_{3}\gamma_{6}\right)}{\gamma_{2}\gamma_{5}+\gamma_{3}}$$

#### MR

To investigate what the Wald Ratio (${\hat{\beta }}_{\mathrm{MRk}}$) using $X_{k}$ as exposure is estimating in our setting, we need to calculate the effect of G on Y, and the effect of G on $X_{k}.$ In our example, we only have two occurrences of the exposure, so *k *=* *0 or 1. For examples with the exposure in continuous time, see the Supplementary material (available as Supplementary data at *IJE* online). We now derive the expressions for what is estimated with exposures $X_{0}$ and $X_{1}$ using the usual Wald Ratio.

The effect of G on Y is:

(3) $$\beta_{GY}=\gamma_{1}\left(\gamma_{2}\gamma_{4}+\gamma_{2}\gamma_{5}\gamma_{6}+\gamma_{3}\gamma_{6}\right)$$

The effect of G on $X_{0}$ is:

(4) $$\beta_{{GX}_{0}}=\gamma_{1}\gamma_{2}$$

The effect of G on $X_{1}$ is:

(5) $$\beta_{{GX}_{1}}=\gamma_{1}\left(\gamma_{2}\gamma_{4}+\gamma_{2}\gamma_{5}+\gamma_{1}\gamma_{3}\right)$$

The Wald Ratio MR estimand with $X_{0}$ as a single exposure is given by Equation (3)/Equation (4):

(6) $$\beta_{{MR}_{0}}=\frac{\gamma_{1}\left(\gamma_{2}\gamma_{4}+\gamma_{2}\gamma_{5}\gamma_{6}+\gamma_{3}\gamma_{6}\right)}{\gamma_{1}\left(\gamma_{2}\right)}=\frac{\left(\gamma_{2}\gamma_{4}+\gamma_{2}\gamma_{5}\gamma_{6}+\gamma_{3}\gamma_{6}\right)}{\left(\gamma_{2}\right)}$$

The Wald Ratio MR estimand $\beta_{{MR}_{0}}$ in Equation (6) is equal to the effect on Y of changing the liability L such that it raises $X_{0}$ by one unit in expectation in Equation (1), and hence estimates $\beta_{L_{0}}$, the liability effect of $X_{0}$ on Y.

The Wald Ratio MR estimand with $X_{1}$ as a single exposure is given by Equation (3)/Equation (5):

(7) $$\beta_{{MR}_{1}}=\frac{\gamma_{1}\left(\gamma_{2}\gamma_{4}+\gamma_{2}\gamma_{5}\gamma_{6}+\gamma_{3}\gamma_{6}\right)}{\gamma_{1}\left(\gamma_{2}\gamma_{5}+\gamma_{3}\right)}=\frac{\left(\gamma_{2}\gamma_{4}+\gamma_{2}\gamma_{5}\gamma_{6}+\gamma_{3}\gamma_{6}\right)}{\left(\gamma_{2}\gamma_{5}+\gamma_{3}\right)}$$

The Wald Ratio MR estimand in Equation (7) is equal to the effect on Y of changing the liability *L* resulting in a one-unit change in $X_{1}$ in Equation (2), and hence estimates $\beta_{L_{1}}$ the liability effect of $X_{1}$ on Y. Importantly, both liability effects include effects through the other exposure X.

MR with a single liability L can therefore only examine whether there is evidence for a causal effect of some measure of the exposure (at some time point in the period in which the liability L operates) on the outcome, not which part of the exposure trajectory is causal. This is because the liability L affects all the exposures jointly in the period in which it operates, so estimating the effect on Y of changing the liability does not give information about which of the exposures caused by the liability cause(s) the outcome. It does not matter whether genotype–exposure associations are time-varying or time-invariant; the null hypothesis tested using MR (which does not require the parametric assumptions, only the structural assumptions) is that the liability L does not cause the outcome, i.e. there is no part of the trajectory that causes the outcome. If the liability does not cause the outcome, a null effect will be correctly detected using MR.^6^ The Wald Ratio MR estimand based on the single exposure measurement $X_{k}$, the *liability effect*, is the effect of increasing L such that $X_{k}$ increases by one unit without holding the other X fixed. We extend this to an outcome measured at multiple time points in the Supplementary material (available as Supplementary data at *IJE* online).

### Simulation approach

We describe our simulation approach within the ADEMP framework.^29^

#### (A)ims

The aim of the simulation was to illustrate the causal effect estimates described above.

#### (D)ata-generating mechanisms

**Figure 3:**
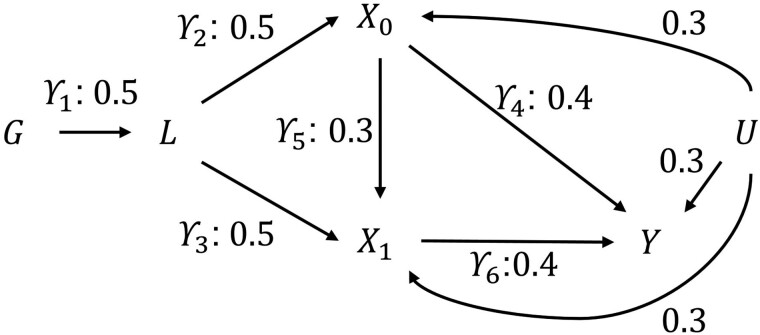
Simulated parameters G, genotype; L , liability; $X_{0}$, exposure measured at time 0; $X_{1},\;\mathrm{exposure}\;\mathrm{measured}\;\mathrm{at}\;\mathrm{time}\;1;\;Y,\;\mathrm{outcome};\;U$, confounder.

We simulated data for 10 000 hypothetical individuals ($n_{\mathrm{obs}}\;=\;10\;000$), representing a genotyped cohort sample with exposure occurring at two time points ($t_{0}{,t}_{1}$). Let G represent the genotype of individuals simulated as a single variant (effect alleles* *=* *0,1,2) with minor allele frequency set to 0.2 and genotype drawn from this with a binomial distribution. We simulate a liability L underlying a time-varying exposure ($X_{k}$) that occurs on two occasions k (*k *=* *0,1), an outcome that occurs once (Y) and a time-invariant confounder (U) of exposure and outcome variables. Random measurement error was simulated for all variables except the genetic instrument. Associations of the exposure and outcome with the unobserved confounder ($\alpha_{0},\;\alpha_{1},\;\alpha_{Y}$) were set at 0.3. For simplicity, the association between the liability and the confounder $\left(\alpha_{L}\right)$ was set to 0. Base parameters were set as follows: $\gamma_{1}\;$=* *0.5 $\gamma_{2}\;$=* *0.5; $\gamma_{3}\;$=* *0.5; $\gamma_{4}\;$=* *0.4; $\gamma_{5}\;$=* *0.3; and $\gamma_{6}\;$=* *0.4 (Figures 2 and 3). One by one all base parameters except $\gamma_{1}$ were set to zero to investigate the change in coefficient estimated. This allowed us to interrogate differential (i) strength of the genetic instrument; (ii) time-varying genetic associations; (iii) exposure effects on the outcome(s); and (iv) confounding effects. Note that the value of the liability effects for the exposures will not remain constant but will change depending on the base parameters. Results are presented for 1000 replications of each simulation. All data were generated within Stata. The programme code used to run the simulations is available at https://github.com/timtmorris/time-varying-MR and can be used to vary all parameters.

#### (E)stimands

We estimate the liability effect $\beta_{L_{k}}$, the effect of increasing L such that $X_{k}$ increases by one unit, on Y by MR using the Wald Ratio and the standard error (SE) of this estimate in our simulations.

#### (M)odel

We assume the linear structural equation model in Figure 3.

#### (P)erformance measures

We used three performance measures to assess the estimates in our simulations: the mean of the estimates of the liability effects for $X_{0}$ and $X_{1}$, the mean of the SEs of the liability effects for $X_{0}$ and $X_{1}$ across 1000 replications, and the deviation (and SE) of the estimates of the liability effects for $X_{0}$ and $X_{1}$ from their expectation given the model parameters.

## Results

### Simulations

Simulations illustrate that the Wald Ratio MR estimator correctly recovers the liability causal effect in all scenarios with a time-varying exposure, even where time-varying genetic associations existed (Table 1). The estimate of the liability effect of $X_{0}$ on Y is different to that of $X_{1}$ on Y. This is because MR is estimating the effect of L on Y rather than the effect of $X_{k}$ itself, i.e. the change in L required to raise $X_{0}$ by one unit is 2 (=* *1/0.5), whereas the change in L required to raise $X_{1}$ by one unit is 1.54 (=* *1/0.65). Non-zero estimates are recovered for the liability effect of $X_{0}$ on Y even when there is no direct path from $X_{0}$ to Y (i.e. $\gamma_{4}\;=\;0$). This non-zero coefficient arises because MR estimates the causal effect of changing the liability such that the exposure measured at time $t_{0}$ is one unit higher, not the effect of a one-unit change in $X_{0}$ whilst holding $X_{1}$ constant. Similarly, there is a non-zero estimate for the liability effect of $X_{1}$ on Y even if there is no direct effect from $X_{1}$ to Y (i.e. $\gamma_{6}\;=\;0$), because the change in liability causing the change in $X_{1}$ will have also changed $X_{0}$. It does not matter when the exposures are measured with respect to the outcome (provided that earlier exposures influence the outcome); non-zero effects of $X_{1}$ on Y will be correctly estimated even if the exposure is measured after the outcome. Note that the MR estimates in Table 1 do not correspond to the estimates of the direct effects of $X_{0}$ or $X_{1}$ on Y except for the cases in which the liability has no effect on $X_{0}$ or either $X_{0}$ or $X_{1}$ has no direct effect on Y. Where there are more than two exposure measurements, all direct effects on the outcome except for the exposure under consideration would have to be zero for MR to provide an unbiased estimate of the direct effect of that exposure.

**Table 1:** Estimates, standard errors and bias of the liability effect of a time-varying exposure on an outcome using Mendelian randomization given the parameters in Figure 3

Parameter values	Liability effect of:	
	$X_{0}$ on Y	$X_{1}$ on Y
	[estimate (SE)]	[estimate (SE)]
	[Bias (SE)]	[Bias (SE)]
Base parameters in directed acyclic graph	0.92 (0.042)	0.71 (0.031)
$\gamma_{2}=0$	–6.93 (57 811.605)	0.4 (0.041)
	7.448 (24.217)	0.001 (0.001)
$\gamma_{3}=0$	0.52 (0.041)	1.76 (0.207)
	–0.001 (0.001)	–0.026 (0.006)
$\gamma_{4}=0$	0.52 (0.042)	0.4 (0.028)
	0.002 (0.001)	–0.001 (0.001)
$\gamma_{5}=0$	0.8 (0.042)	0.8 (0.042)
	0 (0.001)	0.001 (0.001)
$\gamma_{6}=0$	0.4 (0.037)	0.31 (0.031)
	0.002 (0.001)	0.001 (0.001)
U=0	0.92 (0.041)	0.71 (0.03)
	0 (0.001)	0 (0.001)

Bias presented as ‘0.000’ where –0.001 < mean bias < 0.001. SE, standard error.

MR recovers liability effects even where simulations are extended to include outcome–exposure feedback effects or reverse confounding (Supplementary material, available as Supplementary data at *IJE* online). Where the liability does not cause exposure during a specific time period (e.g. a genotype may only cause weight gain after puberty), weak instrument bias may affect estimates of the effect of the change in liability required to increase exposure during that period by one unit (Table 1, where $\gamma_{2}\;=\;0$).^30^ This bias is smaller for later measures of exposure (Table 1, where $\gamma_{3}\;=\;0$) because genetic effects here can operate via earlier occurrences.

Cross-sectional total effects estimated using linear regression are biased even where unobserved confounding from U is absent due to confounding by the liability *L* that underlies the repeat measures of exposure (Supplementary material, available as Supplementary data at *IJE* online).

### MR of body mass index measured at different ages on systolic blood pressure

We used two-sample MR to estimate the liability effect of body mass index (BMI) at different ages on systolic blood pressure (SBP) using the SNP rs9939609 located in the fat mass and obesity-associated gene (FTO). Note that this single SNP approach prohibited standard two-sample MR sensitivity analyses but provided a suitable proof of concept. We estimated FTO–BMI associations from a study using data from the 1958 National Survey of Health and Development British cohort in which BMI was measured on 11 occasions between ages 2 and 53* *years (*n* = 2479) by Hardy *et al*.^21^ We estimated FTO–hypertension associations from a study of Danish individuals in the Copenhagen General Population Study with mean age 57.6* *years [standard deviation (SD): 13.49] by Timpson *et al.* (*n* = 37 027), thus ensuring no sample overlap.^31^ All associations were consistent with the study of individuals in the Rotterdam Study (*n* = 5123) by Labrecque and Swanson.^25^

**Figure 4:**
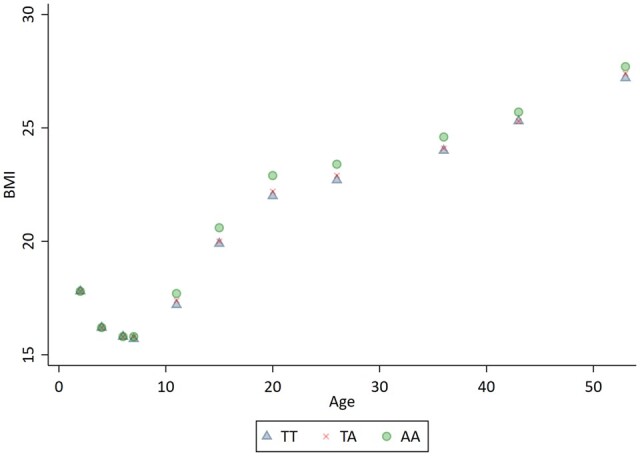
Linear prediction of mean body mass index at different ages from additive genetic models by FTO rs9939609 genotype FTO, fat mass and obesity-associated gene. AA: two risk variant; AT: one risk variant; TT: zero risk variants.

MR results from these SNP–exposure and SNP–outcome associations varied greatly depending on when the exposure was measured (Table 2). This variation in results does not invalidate MR,^25^ but is expected because the effect of genotype on exposure varied over age (Figure 4). The interpretation of the MR estimate is with respect to the underlying liability. So, from Table 2, the effect of changing the liability such that BMI increases by one SD unit in expectation at age 11 years would be to increase mid-life blood pressure by 6.08 mmHg (SE: 2.32 mmHg). The effect of changing the liability such that BMI increases in expectation by one SD unit at age 53 years would be to increase mid-life blood pressure by 12.78 mmHg (SE: 7.77 mmHg). Although these are different, their consistency can be verified by examining Figure 4, and the effect of genotype on BMI at different ages shown in Table 2—the effect of a one-unit change in genotype (which would equate to a change in liability) on measured BMI z-score is twice as large at age 11 years as at age 53 years.

**Table 2:** Results from Mendelian randomization with time-varying exposures for the causal effect of body mass index z-score on systolic blood pressure using rs9939609

Age (years)	SNP–exposure association (SE)	MR estimate (SE)	Association of G with a one-unit SD difference in BMI (SE)	Liability exposure difference equivalent to a one-unit SD difference in exposure at age 20 years (SE)
2	0.013 (0.033)	49.56 (127.47)	78.7 (201.4)	0.09 (0.23)
4	0.019 (0.030)	32.69 (51.68)	51.9 (81.1)	0.14 (0.22)
6	0.0035 (0.031)	178.63 (1546.5)	283.5 (2453.9)	0.025 (0.22)
7	0.043 (0.030)	14.79 (11.18)	23.5 (16.8)	0.30 (0.23)
11	0.104 (0.031)	6.08 (2.32)	9.7 (2.85)	0.73 (0.27)
15	0.107 (0.031)	5.88 (2.23)	9.3 (2.71)	0.76 (0.28)
20	0.141 (0.031)	4.46 (1.46)	7.1 (1.56)	Reference
26	0.097 (0.030)	6.46 (2.52)	10.3 (3.13)	0.69 (0.26)
36	0.070 (0.029)	9.0 (4.30)	14.3 (5.87)	0.50 (0.23)
43	0.043 (0.028)	14.73 (10.30)	23.4 (15.33)	0.30 (0.21)
53	0.049 (0.027)	12.78 (7.77)	20.3 (11.31)	0.35 (0.21)

SNP–exposure associations taken from *Hardy et al.*^21^; SNP–outcome association taken from *Timpson et al.*^31^ Standard errors for ratio estimates were computed using the formula in Burgess *et al.*^32^ ignoring covariance between SNP–exposure effects at different ages.

SNP, single-nucleotide polymorphism; SE, standard error; MR, Mendelian randomization; BMI, body mass index. The SNP–outcome association from Timpson *et al.*^31^ was 0.63 (SE: 0.153).

MR estimates obtained on different occasions can be converted to the same liability scale provided that SNP–exposure associations on these occasions can be estimated. This conversion can be made by multiplying the Wald Ratio estimate by the SNP–exposure association at its age divided by the SNP association at another (target) age. Taking FTO–BMI and FTO–hypertension associations from the Hardy *et al.* and the Timpson *et al.* studies (Table 2), multiplying the age 53 years estimate (12.78 mmHg per SD of BMI) by the SNP–exposure association at age 20 years (0.1412) divided by the SNP–exposure association at age 53 years (0.0493) gives us the liability effect of BMI on SBP at age 20 years of 4.46 $\left(4.46\;=\;12.78\;\times \;\frac{0.0493}{0.1412}\right)$. The third column of Table 2 shows the association of G with a one-unit SD change in BMI at each age. This varies due to variation in SNP–exposure associations at different ages. The last column shows the exposure difference at specific ages that corresponds to a liability-induced increase in BMI of one SD unit at age 20 years. This information helps to interpret the differing MR estimates and could be used to conduct a Genomewide Association Study (GWAS) meta-analysis in which the exposure was measured at different ages in different studies.

## Discussion

Here we have clarified that MR (using the Wald Ratio estimator) using only one measure of an exposure that varies over time estimates the causal effect of the liability that underlies the exposure. That is, MR applied to exposures that vary over time estimates the causal effect of the underlying liability rather than the causal effect of the exposure as it manifests at a given measurement occasion. Although the effect of liability on outcome is the estimand that can be estimated using MR, as the genotype acts on the liability rather than a specific exposure, the liability is unobserved, so we must estimate its effect via the measured exposures. The MR estimate of the liability effect does not require time-invariant genotype–exposure associations under the assumptions that the structural model is linear and additive, providing that the instruments are valid instruments for the underlying liability.^11^^,^^25^ There is also no assumption that the liability L should have the same direction of effect on exposure at all time points.^33^ We do not suggest that the construct of a 1D liability is suitable for every situation with time-varying exposure, but when it is, then the present paper gives an interpretation.

We have demonstrated that MR estimates the causal effect of a change in liability L that results in an expected one-unit change in exposure $X_{t}$. MR with a single genetic proxy of liability cannot in general be used to infer the direct or total effect of an exposure at a specific point in time, or to draw inferences about changes in exposure trajectories different to the one that L causes (e.g. the effect of increasing exposure by one unit at all time points).^25^ Results from MR conducted at different exposure time points will necessarily differ where time-varying gene–exposure associations exist. However, this does not invalidate MR as previously argued,^25^ but highlights that it is testing the effect of the liability L on outcome.^34^ Estimation of time-specific causal effects (i.e. *what is the direct or total effect of the exposure at a given timepoint on the outcome*) will usually not be possible with instruments for one liability that affects all periods of exposure, in the absence of other information.

We proposed a new definition of the causal effect estimated by MR using one measure of a time-varying exposure and one liability, the ‘liability effect’, as *the causal effect of changing the liability such that one particular measurement of exposure would be one unit higher at a given time, and all other occurrences of the exposure would change accordingly*. The estimated liability causal effect will differ in size if the exposure is measured at a different time point, but the estimates will be consistent with the underlying trajectory of exposure induced by the SNP as shown in Table 2. Although the FTO trajectories from the study by Hardy *et al.*^21^ study will likely differ from those in larger studies,^35^ these have been used for illustrative purposes as they cover a broad range of ages. Our interpretation differs from that previously suggested by Labrecque and Swanson^25^ in that it rests upon a liability caused by a specific genotype. Labrecque and Swanson demonstrated that MR is sensitive to age-related variation in SNP–exposure associations and we have demonstrated that these differences are a necessary component of time-varying exposures. Our assumption that genotype may act, through liability L, upon the whole lifecourse exposure trajectory^36^ rather than a single exposure measurement is supported by studies demonstrating time-varying genetic associations.^15–20^

It may seem counter-intuitive to use the instrument to describe the causal effect to be estimated, e.g. we are estimating the effect of the liability that is associated with a given SNP. So if a one-unit increase in SNP causes an increase in exposure at time $t_{0}$ of one unit and an increase in exposure at time $t_{1}$ of two units, then the gene–environment equivalence means that the MR estimate of the effect of this liability on outcome is the same as the effect of any intervention that raised the exposure by one unit at *t*_0_ and two units at time *t*_1_. The underlying point is that we can only examine the effect of a liability that has an instrument associated with it. For example, should an analyst wish to estimate the effect of increasing X by one unit at all time points, an instrument that has a constant effect on exposure over the lifecourse would be required. If the interest is in a liability that causes X to double every 10 years, then an instrument that has this (or a proportional) effect is required. Genetic variants may better relate to relative than absolute differences in some phenotypes and where this is the case, transformations of phenotypes may be considered to demonstrate more consistent effect sizes throughout the lifecourse. Here, MR effects would be consistent over time.

There are thus two consequences of our results. First, if the aim is to estimate the effect of a specific liability, then the researcher needs to find an instrument for that liability. This is no different to any other situation in which some desired exposures cannot be instrumented genetically (e.g. it is hard to imagine a valid genetic instrument for cycling to work). Second, interpretation of an MR of an exposure that varies over time is with respect to the liability for that exposure that is induced by the given genotype. Thus, interpretation of an MR estimate of a time-varying exposure requires knowledge of the liability induced by the genotype. This in turn means that if several genotypes have different effects on the liability for exposure (e.g. one increases exposure during early ages and has a constant effect after age 20 years and another decreases exposure during early ages and has a constant effect after age 20 years) but the same effect on outcome, this would imply that it was the exposure after age 20 years that was important for this outcome. If there are SNPs associated with different liabilities in this way, then multivariable MR (MVMR)^28^ could be formally used to estimate the effects of the liability for exposure before and after age 20 years.

Our simulations also illustrate that MR is not biased with respect to the liability effect by longitudinal exposure mediation, where earlier exposure measures cause later exposure measures. Again, it is not possible to draw inferences on the timing of causal effects because it is the effect of the liability that is being estimated, which goes through exposure at all time points whether measured or not. An effect of the exposure on the outcome could still be observed even if the exposure is measured later than the outcome, as it is the estimated effect of the liability to the exposure on the outcome that will be estimated. Similarly, if an outcome affects a later measurement of exposure, an investigator will not incorrectly conclude that the outcome causes the exposure, but they may incorrectly conclude that exposure at *a given age* causes an outcome. With one liability for an exposure, a cumulative effect of exposure will be indistinguishable from an effect of exposure only during specific time periods; as stated before, one can only say that some part of the exposure trajectory is causal, but not *which* part.^6^ The lack of the ability to determine causal effects at specific time points complicates comparisons between MR and randomized–controlled trials (RCTs). In an RCT, the timing of exposure (intervention) can be modified (e.g. an intervention can be given at any age, or any stage in a disease time course, as specified by the design of the study) and an intervention will have a specific effect on the subsequent pattern of exposure, e.g. randomization to a weight-loss intervention at age 40 years might lower BMI by one unit at age 43 years, but by age 53 years the two arms of the trial could have the same average BMI. This RCT would thus estimate a different effect on outcome than the MR estimate of the liability effect at age 43 years using the FTO genotype shown in Table 2.

Although we investigated a single SNP, this interpretation of time-varying MR can in principle be extended to multiple SNPs if they all act on the same underlying liability to exposure (L, in Figure 1). It is however highly unlikely that any two SNPs will induce the same liability and thus the same exposure trajectory (non-causal SNPs that tag the same causal variant would not be seen to *produce* the trajectory). If different SNPs have differing time-varying associations with the exposure, then their estimated liability effects of exposure on outcome will differ. Future studies should assess heterogeneity between groups of SNPs with repeat measures of exposure to assess the consistency of trajectories of exposure.^11^ Assuming multiple liabilities through MVMR may allow investigators to more reliably test hypotheses about exposures during different time periods.^37^^,^^38^ For example, a recent MR study using multiple instruments with different effects on early (age 10 years) and later life (age 57 years) BMI could draw inferences about the different contributions of liability for BMI at ages 10 and 57 years.^15^ An MR study with a single instrument (or multiple instruments acting on the same liability) could only draw inferences on the whole time period acted on by that liability.

Our liability effect and the Wald Ratio estimator rely on a linear, additive structural model, but do not make any assumptions about the timing of how exposure affects the outcome. For example, the exposure may act cumulatively on the outcome, may have sensitive or critical periods^39^ or may have different effects depending on its proximity to the outcome window. If the mechanism of exposure is known, then the appropriate summary of exposure could be derived and used in MR, e.g. using cumulative exposure or functional principal component analysis to summarize trajectories of exposure.^40^ Further longitudinal genetic studies that investigate time-varying genetic associations with exposures are therefore required to better triangulate causal evidence.^41^

This framework of an effect of an underlying liability can be extended from multiple measures of the same phenotype to measures of different phenotypes. However, in univariable MR, the assumption would be that instruments only act on a common liability and do not have any direct effects on different phenotypes. To estimate separate direct effects on two phenotypes, one would need to use separate liabilities within an MVMR framework.^28^

The key aspect when interpreting MR results from time-varying exposures is to consider the underlying liability for a specific exposure trajectory. We have demonstrated this using two exposure occasions for simplicity, but the result holds when the liability is extended across measures of X in continuous time (Supplementary material, available as Supplementary data at *IJE* online). MR with a genetic instrument using an exposure measured at a single time point provides an unbiased estimate of the causal effect of moving the liability L (as induced by the instrument) such that the exposure at the single time point would be expected to increase by one unit. Care must be taken in interpretation of the results of MR analyses using a single measure of a time-varying exposure, as temporal effects cannot be inferred in the presence of a genetic instrument obtained from a single time point. It is important for future research to consider the exposure trajectories for every genetic instrument used.^25^

## Ethics approval

Ethics approval was not required for this study; only publicly available summary data were used.

## Supplementary Material

dyac136_Supplementary_Data

## Data Availability

The summary data and all code used in this paper are available at https://github.com/timtmorris/time-varying-MR.
