# Supplementary material for: Interpretation of Mendelian randomization using a single measure of an exposure that varies over time
Source: Int J Epidemiol. 2022 Jul 15;51(6):1899–909. doi: 10.1093/ije/dyac136 (PMC9749705; doi:10.1093/ije/dyac136)
Supplement: dyac136_Supplementary_Data [file dyac136_supplementary_data.docx]

# Supplementary material to:

**Interpretation of Mendelian randomization using a single measure of an exposure that varies over time.**

Tim T Morris * ^1,2^, Jon Heron^1,2^, Eleanor Sanderson^1,2^, George Davey Smith^1,2^, Vanessa Didelez^3,4^, Kate Tilling^1,2^.

1. MRC Integrative Epidemiology Unit at the University of Bristol, BS8 2BN, United Kingdom.
2. Population Health Sciences, Bristol Medical School, University of Bristol, Barley House, Oakfield Grove, Bristol, BS8 2BN, United Kingdom.
3. Leibniz Institute for Prevention Research and Epidemiology – BIPS, Bremen, Germany.
4. Department of Mathematics and Computer Science, University of Bremen, Germany.

## Section 1: Time-varying MR extended to continuous exposure measurements

### Liability effect

We could define a lifetime effect of $X_{k}$ on $Y$ as the change in $Y$ from changing the entire trajectory of $X$ such that $X_{k}$ is raised by one unit. However, this is not a uniquely defined estimand – it does not specify how the trajectory of $X$ must be changed, only that the change must be compatible with a one unit rise in $X_{k}$. For example, the definition provided by Labreque and Swanson, of a constant one unit increase in $X$ at all times, would be a lifetime effect. Other possible estimands would include the effect of changing just $X_{k}$ by one unit, whilst keeping all other parts of the trajectory of $X$ constant, or the effect of increasing $X_{k}$ and all subsequent values of $X$ by one unit. In a practical example, the key is to identify instruments for an effect of interest, e.g. by examining the time-varying effect of all instruments for a given exposure.

We define an alternative measure of a lifetime effect as estimated by MR with a specific SNP within the context of time-varying exposures; the “liability effect” of moving the entire exposure history such that exposure at time $t$ increases by one unit. This differs from the lifetime effect provided by Labreque and Swanson, but our definition will be equivalent to theirs under the condition that the association between the SNP and exposure is constant over time. We have an outcome $Y$ which is measured only at time $T$, and an exposure $X$ which occurs in continuous time, where $X_{t}$ is the value of $X$ at time $t$.

We define the liability causal effect on Y of shifting the liability $L$ by one unit as $\beta_{L}.$We define the liability causal effect on Y of shifting the liability $L$ (and thus the entire exposure trajectory) such that the expectation of $X_{k}$ at time $k$ increases by one as $\beta_{L_{k}}$. As in Figure 1, we have a genetic instrument $G$ that acts on the liability $L$, which then causes $X_{t}.$We define the instantaneous effect of $X_{t}$ on $Y$ by $\gamma_{t}$; the effect of $L$ on $X_{t}$ by $\beta_{Lt}$; and the effect of time on $X_{t}$ by $\beta_{0t}$:

$${L=\alpha_{0}+\alpha}_{g}G+\varepsilon_{L}$$

$$X_{t}=\beta_{0t}+\beta_{1t}L=\beta_{0t}+\beta_{1t}\left( {\alpha_{0}+\alpha}_{g}G \right)+\varepsilon_{X}$$

The expectation of $Y$ conditional on the liability being set to $l_{0}$ is:

$$E\left( Y | do\left( l_{0} \right) \right)=\int_{0}^{T} \gamma_{t}(\beta_{0t}+\beta_{1t}l_{0}) dt$$

The expectation of $Y$ conditional on the liability being set to $l_{0}+1$ is:

$$E\left( Y | do\left( l_{0}+1 \right) \right)=\int_{0}^{T} \gamma_{t}(\beta_{0t}+\beta_{1t}{(l}_{0}+1)) dt$$

Thus the effect on $Y$ of increasing the liability by one unit, from $l_{0}$ to $l_{0}+1$, is:

$$E\left( Y | do\left( l_{0}+1 \right) \right)-E\left( Y | do\left( l_{0} \right) \right)=\int_{0}^{T} \gamma_{t}\left( \beta_{0t}+\beta_{1t}{(l}_{0}+1) \right)dt-\int_{0}^{T} \gamma_{t}\left( \beta_{0t}+\beta_{1t}l_{0} \right)dt=\int_{0}^{T} \gamma_{t}\left( \beta_{1t} \right)dt$$

We now consider the liability effect of $X_{k}$, i.e. the effect on $Y$ of changing the liability such that $X_{k}$ increases by 1. This is the expected value of $Y$ conditional on the liability being set to $l_{0}+\frac{1}{\beta_{1k}}$, which is:

$$E\left( Y | do\left( l_{0}+\frac{1}{\beta_{1k}} \right) \right)=\int_{0}^{T} \gamma_{t}\left( {\beta_{0k}+\beta}_{1t}\left( l_{0}+\frac{1}{\beta_{1k}} \right) \right)dt$$

Therefore, the liability effect of $X_{k}$ on $Y$ (the effect of changing the liability $L$ such that $X_{k}$increases by 1) is given by $\beta_{L_{k}}$

|  | $\beta_{L_{k}}=E\left( Y \vert do\left( l_{0}+\frac{1}{\beta_{Lk}} \right) \right)-E\left( Y \vert do\left( l_{0} \right) \right)=\int_{0}^{T} {\frac{1}{\beta_{1k}} \gamma_{t}}\beta_{1t} dt=\frac{1}{\beta_{1k}}\int_{0}^{T} \gamma_{t}\beta_{1t} dt$ | (A1) |
| --- | --- | --- |

We now consider the effect on both $X$ and $Y$ of changes in $G$. The structural model for $X_{t}$ when $G=g$ is:

$$E\left[ X_{t}|do(g) \right]=\beta_{0}+\beta_{Lt}({\alpha_{0}+\alpha}_{g}g)$$

Using this in the structural model for $Y|G$

$$E\left[ Y|do(g+1)]-E[{Y|do(g)]} \right]=\int_{0}^{T} \gamma_{t}(\beta_{0t}+\beta_{1t}\left( {\alpha_{0}+\alpha}_{g}g \right)) dt-\int_{0}^{T} \gamma_{t}(\beta_{0t}+\beta_{1t}\left( a_{0} \right))dt$$

Which reduces to:

|  | $E\left[ Y\vert do(g+1)]-E[{Y\vert do(g)]} \right]=\int_{0}^{T} \gamma_{t}(\beta_{1t}\left( \alpha_{g} \right)) dt$ | (A2) |
| --- | --- | --- |

The structural model for $X_{k}$ is:

|  | $E\left[ X_{k}\vert do(g+1) \right]-E\left[ X_{k}\vert do(g) \right]=\beta_{0k}+\beta_{1k}\left( {\alpha_{0}+\alpha}_{g} \right)-{(\beta}_{0k}+\beta_{1k}\left( a_{0} \right))=\beta_{1k}(\alpha_{g})$ | (A3) |
| --- | --- | --- |

The Wald Ratio estimator is given as the change in the outcome for a given change in the instrument, divided by the change in the exposure for the same change in the instrument. Dividing the reduced form (A2) by the genetic effect measured at time $k$ (A3) we obtain:

$$\frac{E\left[ Y|do(g+1)]-E[{Y|do(g)]} \right]}{E\left[ X_{k}|do(g+1) \right]-E\left[ X_{k}|do(g) \right]}=\frac{\int_{0}^{T} \gamma_{t}(\beta_{1t}\left( \alpha_{g} \right)) dt}{\beta_{1k}(\alpha_{g})}=\frac{1}{\beta_{1k}}\int_{0}^{T} \gamma_{t}\beta_{1t} dt$$

This is the same as the causal effect in equation (A1), showing that MR can be interpreted as the liability effect of genetically inducing an increase in exposure by one unit at time $k$.

## Section 2: MR in the presence of reverse causation

### Liability effect

Here we consider the case where we have a time-varying outcome (with measures $Y_{0}$ and $Y_{1}$) and an earlier measure of the outcome causes a later measure of the exposure (Figure S1). We show that the MR estimate is an unbiased estimate of the causal effect of a change in liability $L$ such that there is a one unit change in $X$ at the given time.

#### Figure S1: DAG showing the liability in the context of two exposures and two outcomes. $\boldsymbol{G}$, genotype; $\boldsymbol{L}$, liability; $\boldsymbol{X}$, exposure; $\boldsymbol{Y}$, outcome; $\boldsymbol{U}$, confounder. Subscripts denote timepoint.

$$\Upsilon_{1}$$

$$\Upsilon_{2}$$

$$\Upsilon_{3}$$

$$\Upsilon_{5}$$

$$\Upsilon_{4}$$

$$\Upsilon_{6}$$

$$\Upsilon_{9}$$

$$\Upsilon_{7}$$

$$\Upsilon_{8}$$

For a given $L=l_{0}$, then the two outcomes are given by:

$$Y_{0}=l_{0}\left( \gamma_{2}\gamma_{4} \right)$$

And

$$Y_{1}=l_{0}\left( \gamma_{2}\gamma_{4}\gamma_{9}+\gamma_{2}\gamma_{4}\gamma_{8}\gamma_{6}+\gamma_{2}\gamma_{5}\gamma_{6}+\gamma_{2}\gamma_{7}+\gamma_{3}\gamma_{6} \right)$$

A one unit increase in expectation of $X_{0}$ occurs because there is an increase from $l_{0}$ to $l_{0}+\frac{1}{(\gamma_{2})}$

The effect on $Y_{0}$ of a change in liability $L$ such that there is a one unit change in expectation of $X_{0}$ is therefore given by:

|  | $\beta_{{LY_{0}X}_{0}}=\frac{1}{\gamma_{2}}\left( \gamma_{2}\gamma_{4} \right)$ | (B1) |
| --- | --- | --- |

The effect on $Y_{1}$ of a change in liability $L$ such that there is a one unit change in expectation of $X_{0}$ is therefore given by:

|  | $\beta_{{LY_{1}X}_{0}}=\frac{1}{\gamma_{2}}\left( \gamma_{2}\gamma_{4}\gamma_{9}+\gamma_{2}\gamma_{4}\gamma_{8}\gamma_{6}+\gamma_{2}\gamma_{5}\gamma_{6}+\gamma_{2}\gamma_{7}+\gamma_{3}\gamma_{6} \right)$ | (B2) |
| --- | --- | --- |

A one unit increase in expectation of $X_{1}$ occurs because there is an increase in $L$ from $l_{1}$ to $l_{1}+\frac{1}{\left( \gamma_{2}\gamma_{5}+\gamma_{3}+\gamma_{2}\gamma_{4}\gamma_{8} \right)}$

The effect on $Y_{0}$ of a change in liability $L$ such that there is a one unit change in expectation of $X_{1}$ is therefore given by:

|  | $\beta_{{LY_{0}X}_{1}}=\frac{1}{\gamma_{2}\gamma_{5}+\gamma_{3}+\gamma_{2}\gamma_{4}\gamma_{8}}\left( \gamma_{2}\gamma_{4} \right)$ | (B3) |
| --- | --- | --- |

The effect on $Y_{1}$ of a change in liability $L$ such that there is a one unit change in expectation of $X_{0}$ is therefore given by:

|  | $\beta_{{LY_{1}X}_{0}}=\frac{1}{\gamma_{2}\gamma_{5}+\gamma_{3}+\gamma_{2}\gamma_{4}\gamma_{8}}\left( \gamma_{2}\gamma_{4}\gamma_{9}+\gamma_{2}\gamma_{4}\gamma_{8}\gamma_{6}+\gamma_{2}\gamma_{5}\gamma_{6}+\gamma_{2}\gamma_{7}+\gamma_{3}\gamma_{6} \right)$ | (B4) |
| --- | --- | --- |

### Mendelian Randomisation

To calculate the MR estimate of the effect of $X_{k}$ on $Y$ using the Wald Ratio, we need to calculate the effect of $G$ on $Y$, and the effect of $G$ on $X_{k}.$

The effect of $G$ on $Y_{0}$ is:

|  | $\beta_{GY_{0}}=\gamma_{1}\left( \gamma_{2}\gamma_{4} \right)$ | (B5) |
| --- | --- | --- |

The effect of $G$ on $Y_{1}$ is:

|  | $\beta_{GY_{0}}=\gamma_{1}\left( \gamma_{2}\gamma_{4}\gamma_{9}+\gamma_{2}\gamma_{4}\gamma_{8}\gamma_{6}+\gamma_{2}\gamma_{5}\gamma_{6}+\gamma_{2}\gamma_{7}+\gamma_{3}\gamma_{6} \right)$ | (B6) |
| --- | --- | --- |

The effect of $G$ on $X_{0}$ is:

|  | $\beta_{{GX}_{0}}=\gamma_{1}\gamma_{2}$ | (B7) |
| --- | --- | --- |
|  |  |  |

The effect of $G$ on $X_{1}$ is:

|  | $\beta_{{GX}_{1}}=\gamma_{1}\left( \gamma_{2}\gamma_{5}+\gamma_{3}+\gamma_{2}\gamma_{4}\gamma_{8} \right)$ | (B8) |
| --- | --- | --- |
|  |  |  |

The Wald ratio MR estimand with $X_{0}$ as a single exposure is given by (B6)/(B7):

|  | $\beta_{{MRY_{1}X}_{0}}=\frac{\gamma_{1}\left( \gamma_{2}\gamma_{4}\gamma_{9}+\gamma_{2}\gamma_{4}\gamma_{8}\gamma_{6}+\gamma_{2}\gamma_{5}\gamma_{6}+\gamma_{2}\gamma_{7}+\gamma_{3}\gamma_{6} \right)}{\gamma_{1}\left( \gamma_{2} \right)}$ | (B9) |
| --- | --- | --- |

Thus, the MR estimate in (B9) is equal to the effect on $Y_{1}$ of a change in liability $L$ such that there is a one unit change in $X_{0}$ in (B4).

Similar results follow for the MR estimates of the effect on $Y_{1}$ of a change in liability $L$ such that there is a one unit change in $X_{1}$, and on $Y_{0}$ of a change in liability $L$ such that there is a one unit change in $X_{0}$ or $X_{1}$. Thus, where there is reverse causation, the MR estimates are unbiased estimates of the effect of a change in liability $L$ such that $X$ is one point higher at time $k$.

Simulations were repeated for two outcome measurements as shown in Figure S2. This additionally allowed us to interrogate differential effects of earlier exposures on later exposures, and reverse causation from earlier outcome measures on later exposure measures.^26^

### Simulation approach

### (A)ims

The aims of the simulations were to evaluate the accuracy with which MR recovers causal estimates of a time-varying exposure on a time-varying outcome.

### (D)ata-generating mechanisms

We simulated data for 10,000 hypothetical individuals ($n_{obs}=10,000$), representing a cohort sample with genotypic and phenotypic data collected at two time points ($t_{0}{,t}_{1}$). Let $G$ represent the genotype of individuals simulated as a single variant (effect alleles = $0,1,2$) with minor allele frequency (MAF) set to $0.2$ and genotype drawn from this with a binomial distribution. We simulate a time-varying exposure ($X_{k}$) for measurement occasions $k$, an outcome measured twice ($Y_{k}$), and a time-invariant confounder ($U$) of exposure and outcome variables. Random measurement error was simulated for all variables except the genetic instrument. Base parameters were set as follows: $\gamma_{2}$: 0.5; $\gamma_{3}$: 0.5; $\gamma_{4}$: 0.4; $\gamma_{5}$: 0.3; $\gamma_{6}$: 0.4; $\gamma_{7}$: 0.4; $\gamma_{8}$: 0.2; and $\gamma_{9}$: 0.2 (Figure S2) All confounder associations were set to 0.3. One-by-one these base parameters were set to zero to investigate the change in coefficient estimated by MR. This allowed us to interrogate differential (i) strength of the genetic instrument; (ii) time-varying genetic associations; (iii) exposure effects on the outcome(s); and (iv) confounding effects. Note that the value of the unbiased estimate will not remain constant but will change depending on the base parameters. Results are presented for 1,000 replications of each simulation. All data were generated within Stata. The programme code used to run the simulations is available at <https://github.com/timtmorris/time-varying-MR> and can be used to vary all parameters.

### (E)stimands

We assessed the causal effect of $X_{k}$ on $Y_{k}$ and the standard error (SE) of this parameter in our simulations.

### (M)odel

We assess the accuracy of Instrumental Variables (IV) analyses.

### (P)erformance measures

We used three performance measures to assess the estimands in our simulations: the mean of the parameter β, the mean of the parameter SE across 1,000 replications, and the deviation of β from its expectation given the model parameters.

#### Figure S2: Simulated parameters. $\boldsymbol{G}$, genotype; $\boldsymbol{L}$, liability; $\boldsymbol{X}_{\boldsymbol{0}}$, exposure measured at time 0; $\boldsymbol{X}_{\boldsymbol{1}}$, exposure measured at time 1;$\boldsymbol{Y}_{\boldsymbol{0}}$, exposure measured at time 0; $\boldsymbol{Y}_{\boldsymbol{1}}$, exposure measured at time 1; $\boldsymbol{U}$, confounder.

$$\Upsilon_{2}:0.5$$

$$\Upsilon_{3}:0.5$$

$$\Upsilon_{5}:0.3$$

$$\Upsilon_{4}:0.4$$

$$\Upsilon_{6}:0.4$$

$$\Upsilon_{9}:0.2$$

$$\Upsilon_{8}:0.2$$

$$\Upsilon_{7}:0.4$$

$$\Upsilon_{1}:1$$

### MR estimates of time-varying exposures in the presence of reverse causation

Simulations demonstrated that MR recovered the correct causal estimate in the presence of time-varying outcomes with outcome-exposure effects (Table S1). Where the parameter $\gamma_{5}$ was set to zero (i.e. no direct effect of $X_{0}$ on$X_{1}$), a non-zero effect of 0.91 (SE: 0.04) is correctly estimated for the liability effect of $X_{0}$ on $Y_{1}$.

#### Table S1: Betas, standard errors, and bias of the liability effect of a time-varying exposure on a time-varying outcome in the presence of reverse causation using MR. Bias presented as “0.000” where -0.001<mean bias <0.001. Note that the rows present the estimate and bias of the target estimate when each parameter is changed, not the estimate of the parameter itself.

|  |  |  | Liability effect of: | | |
| --- | --- | --- | --- | --- | --- |
|  |  |  | $X_{0}$ on $Y_{0}$ | $X_{0}$ on $Y_{1}$ | $X_{1}$ on $Y_{1}$ |
| Estimated causal effect given base parameters in DAG | | b (se) | 0.4 (0.037) | 1.03 (0.044) | 0.75 (0.03) |
| Estimated causal effect when setting the following parameter to zero: | |  |  |  |  |
|  | $\gamma_{2}$ | b (se) | 1.19 (2260.278) | 0.18 (145000) | 0.4 (0.044) |
|  |  | bias | -0.787 (1.372) | 0.454 (41.284) | -0.002 (0.001) |
|  | $\gamma_{3}$ | b (se) | 0.4 (0.037) | 0.63 (0.043) | 1.67 (0.154) |
|  |  | bias | 0.001 (0.001) | 0.001 (0.001) | -0.008 (0.005) |
|  | $\gamma_{4}$ | b (se) | -0.001 (0.037) | 0.92 (0.044) | 0.71 (0.031) |
|  |  | bias | 0.001 (0.001) | -0.001 (0.001) | 0 (0.001) |
|  | $\gamma_{5}$ | b (se) | 0.4 (0.037) | 0.91 (0.044) | 0.85 (0.04) |
|  |  | bias | -0.001 (0.001) | 0 (0.001) | -0.003 (0.001) |
|  | $\gamma_{6}$ | b (se) | 0.4 (0.037) | 0.48 (0.038) | 0.35 (0.03) |
|  |  | bias | -0.001 (0.001) | 0.001 (0.001) | 0 (0.001) |
|  | $\gamma_{7}$ | b (se) | 0.4 (0.037) | 0.63 (0.044) | 0.46 (0.028) |
|  |  | bias | 0 (0.001) | 0.001 (0.001) | -0.001 (0.001) |
|  | $\gamma_{8}$ | b (se) | 0.4 (0.037) | 1.004 (0.043) | 0.77 (0.033) |
|  |  | bias | 0.001 (0.001) | -0.004 (0.001) | -0.001 (0.001) |
|  | $\gamma_{9}$ | b (se) | 0.4 (0.037) | 0.95 (0.042) | 0.69 (0.029) |
|  |  | bias | 0 (0.001) | 0 (0.001) | -0.001 (0.001) |
|  | U | b (se) | 0.4 (0.035) | 1.03 (0.042) | 0.75 (0.029) |
|  |  | bias | 0 (0.001) | -0.002 (0.001) | -0.001 (0.001) |

## Section 3: Cross-sectional total effects are confounded by genotype

Consider an outcome $Y$ that is caused by two genetically influenced exposures $X_{0}$ and $X_{1}$ (Figure S3). Here, a linear regression model of $Y$ on $X_{0}$ will estimate a biased parameter for the total effect of $X_{0}$ even where there is no unobserved confounding from $U$. This is because the liability underlies the repeat measures of exposure, itself acting as a source of unmeasured confounding between exposure and the outcome. This creates a condition of confounding by common intercept. A linear regression with $Y$ as the dependent variable (outcome) and $X_{0}$ as the independent variable (exposure) therefore estimates the total effect of $X_{0}$ on $Y$, which also includes confounding by $G$. Controlling for the genetic instrument breaks this back door path of confounding in linear regression.

Linear regression estimates applied to time-varying exposures with time-varying genetic effects cannot therefore be interpreted causally, even where unobserved confounding due to traditional sources is not present. There are two circumstances in which this longitudinal confounding by liability may be avoided. First, where every other exposure measure at every other timepoint is conditioned upon, or second, where a causal effect of $X_{k}$ exists *only* at timepoint $k$, and $X$ was measured at this time. It is therefore questionable whether linear regression estimates an informative parameter in the presence of time-varying exposures.

#### Figure S3: DAG showing the liability in the context of two exposures and one outcome. $\boldsymbol{G}$, genetic instrument; $\boldsymbol{L}$, liability; $\boldsymbol{X}_{\boldsymbol{0}}$, exposure measured at time 0; $\boldsymbol{X}_{\boldsymbol{1}}$, exposure measured at time 1; $\boldsymbol{Y}$, outcome; $\boldsymbol{U}$, confounder.

$$\Upsilon_{1}$$

$$\Upsilon_{2}$$

$$\Upsilon_{3}$$

$$\Upsilon_{5}$$

$$\Upsilon_{4}$$

$$\Upsilon_{6}$$

### Simulation approach

### (A)ims

The aims of the simulations were to evaluate the accuracy with which MR recovers causal estimates of a time-varying exposure on a time-varying outcome.

### (D)ata-generating mechanisms

We simulated data for 10,000 hypothetical individuals ($n_{obs}=10,000$), representing a cohort sample with genotypic and phenotypic data collected at two time points ($t_{0}{,t}_{1}$). Let $G$ represent the genotype of individuals simulated as a single variant (effect alleles = $0,1,2$) with minor allele frequency (MAF) set to $0.2$ and genotype drawn from this with a binomial distribution. We simulate a time-varying exposure ($X_{k}$) for measurement occasions $k$, an outcome measured once ($Y$), and a time-invariant confounder ($U$) of exposure and outcome variables. Random measurement error was simulated for all variables except the genetic instrument. Base parameters were set as follows: $\gamma_{2}$: 0.5; $\gamma_{3}$: 0.5; $\gamma_{4}$: 0.4; $\gamma_{5}$: 0.3; and $\gamma_{6}$: 0.4 (Figure S4). All confounder associations were set to 0.3. One-by-one we changed the base parameters for $b_{1}$, $c$ and $u$ to zero to investigate the change in coefficient estimated by linear regression. This allowed us to interrogate (i) time-varying and time-invariant genetic associations; and (ii) confounding effects. Results are presented for 1,000 replications of each simulation. All data were generated within Stata. The program code used to run the simulations is available at <https://github.com/timtmorris/time-varying-MR> and can be used to vary all parameters.

### (E)stimands

We assessed the total effect of $X_{0}$ on $Y$ and the standard error (SE) of this parameter in our simulations.

### (M)odel

We assess the accuracy of linear regression analyses under two approaches: (i) where the genetic instrument is omitted from the model; and (ii) where the genetic instrument is included in the model.

### (P)erformance measures

We used three performance measures to assess the estimands in our simulations: the mean of the parameter β, the mean of the parameter SE across 1,000 replications, and the deviation of β from its expectation given the model parameters.

#### Figure S4: Simulated parameters. $\boldsymbol{G}$, genotype; $\boldsymbol{L}$, liability;$\boldsymbol{X}_{\boldsymbol{0}}$, exposure measured at time 0; $\boldsymbol{X}_{\boldsymbol{1}}$, exposure measured at time 1; $\boldsymbol{Y}$, outcome; $\boldsymbol{U}$, confounder. Parameters in red font are those that were varied.

$$\Upsilon_{2}\text{:}\text{ }0.5$$

$$\Upsilon_{3}\text{:} 0.5$$

$\Upsilon_{5}$*:* $0.3$

$$\Upsilon_{6}\text{:}0.4$$

$$\Upsilon_{4}\text{:}\text{ }0.4$$

$$0.3$$

$$0.3$$

$$0.3$$

$$\Upsilon_{1}\text{:} 0.5$$

*Cross-sectional total effects are confounded by genotype*

Table S2 displays the results of the simulations. Linear regression failed to recover the correct total estimate of $X_{0}Y$ in the presence of unobserved confounding by $U$. Where unobserved confounding by $U$ was absent, linear regression only recovered the correct total estimate of $X_{0}Y$ if the genetic instrument was included in the regression model or there was no confounding by genotype (parameter $\gamma_{3}$ set to zero). This suggests that in the presence of time-varying genetic effects, linear regression will remain biased even where there is no unobserved confounding by traditional sources. Given the complexity of real-world exposure trajectories, this highlights the difficulty of interpreting cross-sectional estimates of a repeat measure exposure.

#### Table S2: Linear regression estimates, standard errors and bias when estimating the total effect of an exposure on an outcome ($\boldsymbol{X}_{\mathbf{0}}\boldsymbol{Y}$) using linear regression.

| *Confounding by U* | *G included in analysis model* | *Parameter set to zero* | *Expected value* | *Estimate (SE)* | *Bias* |
| --- | --- | --- | --- | --- | --- |
| Absent | No | $\gamma_{3}$ | 0.52 | 0.519 (0.010) | 0.0006 (0.0003) |
| Present | No | $\gamma_{3}$ | 0.52 | 0.628 (0.011) | -0.108 (0.0003) |
| Absent | Yes | $\gamma_{3}$ | 0.52 | 0.520 (0.011) | 0.0001 (0.0004) |
| Present | Yes | $\gamma_{3}$ | 0.52 | 0.636 (0.011) | -0.116 (0.0004) |
| Absent | No | $\gamma_{5}$ | 0.4 | 0.429 (0.010) | -0.029 (0.0003) |
| Present | No | $\gamma_{5}$ | 0.4 | 0.535 (0.011) | -0.135 (0.0003) |
| Absent | Yes | $\gamma_{5}$ | 0.4 | 0.400 (0.011) | 0.0003 (0.0003) |
| Present | Yes | $\gamma_{5}$ | 0.4 | 0.515 (0.011) | -0.115 (0.0003) |
| Absent | No | *None* | 0.52 | 0.550 (0.01) | -0.03 (0.0003) |
| Present | No | *None* | 0.52 | 0.655 (0.011) | -0.135 (0.0003) |
| Absent | Yes | *None* | 0.52 | 0.520 (0.011) | 0.0002 (0.0003) |
| Present | Yes | *None* | 0.52 | 0.635 (0.011) | -0.115 (0.0004) |
